# GWAS revealed effect of genotype × environment interactions for grain yield of Nebraska winter wheat

**DOI:** 10.1186/s12864-020-07308-0

**Published:** 2021-01-02

**Authors:** Shamseldeen Eltaher, P. Stephen Baenziger, Vikas Belamkar, Hamdy A. Emara, Ahmed A. Nower, Khaled F. M. Salem, Ahmad M. Alqudah, Ahmed Sallam

**Affiliations:** 1 Department of Agronomy & Horticulture, University of Nebraska-Lincoln, Lincoln, USA; 2 Department of Plant Biotechnology, Genetic Engineering and Biotechnology Research Institute (GEBRI), University of Sadat City (USC), Sadat City, Egypt; 3 Department of Biology, College of Science and Humanitarian Studies, Shaqra University, Qwaieah, Saudi Arabia; 4 Institute of Agricultural and Nutritional Sciences, Martin Luther University Halle-Wittenberg, Betty-Heimann-Str. 3, 06120 Halle (Saale), Germany; 5 Department of Genetics, Faculty of Agriculture, Assiut University, Assuit, 71526 Egypt

**Keywords:** Bread wheat (*Triticum aestivum* L.), Yield, LD, Association mapping, Gene annotation, Breeding programs

## Abstract

**Background:**

Improving grain yield in cereals especially in wheat is a main objective for plant breeders. One of the main constrains for improving this trait is the G × E interaction (GEI) which affects the performance of wheat genotypes in different environments. Selecting high yielding genotypes that can be used for a target set of environments is needed. Phenotypic selection can be misleading due to the environmental conditions. Incorporating information from phenotypic and genomic analyses can be useful in selecting the higher yielding genotypes for a group of environments.

**Results:**

A set of 270 F_3:6_ wheat genotypes in the Nebraska winter wheat breeding program was tested for grain yield in nine environments. High genetic variation for grain yield was found among the genotypes. G × E interaction was also highly significant. The highest yielding genotype differed in each environment. The correlation for grain yield among the nine environments was low (0 to 0.43). Genome-wide association study revealed 70 marker traits association (MTAs) associated with increased grain yield. The analysis of linkage disequilibrium revealed 16 genomic regions with a highly significant linkage disequilibrium (LD). The candidate parents’ genotypes for improving grain yield in a group of environments were selected based on three criteria; number of alleles associated with increased grain yield in each selected genotype, genetic distance among the selected genotypes, and number of different alleles between each two selected parents.

**Conclusion:**

Although G × E interaction was present, the advances in DNA technology provided very useful tools and analyzes. Such features helped to genetically select the highest yielding genotypes that can be used to cross grain production in a group of environments.

**Supplementary Information:**

The online version contains supplementary material available at 10.1186/s12864-020-07308-0.

## Background

Bread wheat (*Triticum aestivum* L.) is the third most important food crop in the world after maize (*Zea mays* L.) and rice (*Oryza sativa* L). To meet the increasing food demand of a growing population, the breeders have focused on the development of cultivars having higher yield and yield stability, and increased resistance/tolerance to biotic and abiotic stresses. Grain yield (GY) is controlled by numerous genes that interact with each other and with the environment [2, 50]. Grain yield is a complex trait that is determined by multiple yield component traits, and each component trait is a quantitative trait controlled or affected by multiple loci [2, 72]. Thus, there needs a detailed genetic dissection of the grain yield trait and its component traits to manipulate the alleles at the relevant loci to the greatest advantage.

F_3:6_ Nebraska winter wheat genotypes are tested in nine environments (8 environments in Nebraska and one in Kansas). Multi-environment yield trials (MEYTs) are used in the last selection cycles to identify superior genotypes in plant breeding programs and to determine where the cultivars are best adapted. This task is difficult due to the frequent presence of GEI.

The GEI reduces the association between phenotype and genotype by reducing heritability, and eventually genetic progress in plant breeding programs. Means across environments are adequate indicators of genotypic performance only in the absence of GEI. If it is present, the use of means across environments ignores the fact that genotypes differ in relative performance in different environments [33]. Analysis of Variance (ANOVA) analysis is not sufficient to provide an understanding of the genotypes or environments that give rise to the interaction [33, 53]. The purpose of MEYTs is not only to classify superior genotypes for the target area, but also to determine if the target area can be divided into mega environments (MEs). Investigation of ME is a requirement for meaningful cultivar evaluation and recommendation [66]. The international Maize and Wheat Improvement Center (CIMMYT) introduced the definition of ME, defined as a broad, not essentially attached area, occurring in more than one country and frequently transcontinental, defined by similar biotic and abiotic stresses, cropping system supplies, customer favorites, and, for convenience, by volume of production [13].

Traditional wheat breeding is mostly built on phenotypic selection which is one of the most important steps for genetic improvement. Every wheat breeder chooses to have an environment at the selection nursery site that will increase the beneficial and minimize the negative aspects of natural selection. In winter wheat breeding, for example, it is common for the selection nursery to be an environment which causes the death of winter tender lines. However, it is blindness, analytical, inefficiency and costs a long time [8]. Fortunately, the hardworking and intelligence of the breeders would have kept signatures in the wheat genome during crop improvement, and this selection signal could be detected using different methods. GWAS should be performed to annotate the signatures in detail, taking into account the selection signal could not be correlated with phenotype [37]. Wheat breeders use single nucleotide polymorphism (SNP) high-density maps to identify genomic regions associated with quantitative traits in biparental mapping experiments or in genome-wide association studies (GWAS) [6, 10, 21, 45, 59, 63, 70]. There are many reports that dissect the effect of genotype, environment, and GEI using linkage mapping effects ([39, 42]; L. [41, 65]). But, the reports on the dissection of GEI using genome-wide association mapping methods are rare [65]. Therefore, the objectives of the present study were to i) study the genetic variation and GEI using the genotype main effects and genotype × environment interaction effects (GGE-biplot analysis) for grain yield for 270 F_3:6_ Nebraska winter wheat genotypes grown in different environments, ii) identify the highest yielding genotypes at the different environments iii) identify marker trait association (MTAs) related to grain yield trait and to dissect GEI using GWAS.

## Methods

### Plant materials

A set of 270 F_3:6_ wheat genotypes (Nebraska Duplicate Nursery, hereafter referred to as DUP2017) which is the preliminary yield trial was derived from 800 to 1000 crosses among Nebraska’s adapted cultivars or experimental genotypes [24]. The parents of these crosses are mainly from wheat breeding programs in the Great Plains, and a few crosses to globally important wheat lines. The breeding lines used in this study were derived from 85 crosses of 800–1000 that were initially made. The pedigree of all 270 genotypes was presented in (Supplementary Table S1).

In wheat growing season 2016/2017, DUP2017 was grown in nine environments [Mead (latitude 41.2286° N, and longitude 96.4892° W), Lincoln, (latitude 40.8136° N, and longitude 96.7026° W) Clay Center (latitude 40.5217° N, longitude 98.0553° W), North Platte (latitude 41.1403° N, and longitude 100.7601° W), Grant, (latitude 40.8430° N, and longitude 101.7252° W) McCook, (latitude 40.1967° N, and longitude 100.6249° W),Sidney,(latitude 41.1448° N, and longitude 102.9774° W) and Alliance (latitude 42.0930° N, and longitude 102.8702° W) in Nebraska, and one location in Kansas (latitude 39.1836° N, and longitude 96.5717° W)]. The experimental layout was incomplete augmented block design with one replication in each location. The incomplete blocks consisted of 27 experimental genotypes and three check cultivars (Goodstreak, Camelot, and Freeman) and there were 10 incomplete blocks per trial. The check cultivars (Goodstreak, Camelot and Freeman) are adapted to diverse ecogeographic regions of Nebraska and by parentage and morphology quite diverse. At all locations, the plots (*N* = 300) were planted at a seeding rate of 54 kg/ha and the plot consisted of five rows of 3 m length with 0.23 m between rows.

### Phenotyping

Grain yield was measured using a combine harvester which harvested all five rows of each plot. At Lincoln and Mead, the harvested grain was stored until dried to room humidity before weighing. At the other locations, the grain was weighed on the combine.

### Genotyping-by-sequencing and SNPs calling

DNA was extracted from the wheat leaves of 2–3 young two-week-old seedlings using BioSprint 96 DNA Plant Kits (Qiagen Valencia, California, USA) following the manufacturer’s instructions. The genotyping-by-sequencing (GBS) was performed as described by Poland et al. [48]. The SNPs were called using Tassel v5.2.40 GBS analysis pipeline with default parameters [12]. The GBS-tags were aligned to the reference genome using Burrows-Wheeler Aligner [36]. The reference genome v1.0 of the ‘Chinese Spring’ genome assembly from the International Wheat Genome Sequencing Consortium (IWGSC) was used in SNP calling. The raw sequence data of the 270 genotypes of the current study along with 6791 other genotypes previously genotyped in our program were combined for SNP calling in order to increase the coverage of the genome and read depth at SNP sites [9, 71]. A set of 200,064 SNPs were resulted from SNP calling. SNPs were removed from the dataset if they were either monomorphic, showed more than 20% missing values, had conflicting calls from SNP or exhibited minor allele frequencies (MAF) of less than 5% [30, 71]. Interestingly, none of our lines (270 F_3,6_) have missing information’s of more than 20%. The GBS data is available in (Supplementary Table S2).

### Statistical analysis

For the field experiments, grain yield was analyzed using methods used for augmented design with replicated check cultivars (augmented incomplete block design). The augmented design is especially useful for statistically controlling spatial variability in large trials (with minimal or no replicates) to assess genotypic effects where seed is often limiting. In the early stages of a breeding program, a plant breeder is faced with evaluating the performance of large numbers of genotypes with limited seed. A general technique for unreplicated designs is the one known as “systematically spaced checks.” In this technique, a standard check(s) genotype is systematically spaced in the trial [25].

The incomplete block consisted of 27 experimental lines and the three check cultivars which were planted in ten incomplete blocks for a total of 300 plots. The liner mixed model was done using this model.

$$ \mathrm{Y}=\mathrm{Check}+\mathrm{Environment}+\mathrm{Iblock}\ \left(\mathrm{Environment}\right)+\mathrm{Genotype}+\mathrm{GXE}+\mathrm{Error} $$

In this model all terms except checks were fit as random effect, and the check was fit as fixed effect. The residual maximum likelihood (REML) implemented in ASREML-R version 4.1 [15]. was used to estimate the variance components and the associated standard errors. The likelihood ratio test using “lrt” function in ASREML-R was used to test significance for each term [16]. The variance component was used also to estimate broad sense heritability using the following formula:

$$ {\mathrm{H}}^2=\mathrm{Var}\left(\mathrm{G}\right)/\left(\mathrm{Var}\left(\mathrm{G}\right)+\mathrm{Var}\ \left(\mathrm{G}\mathrm{XE}\right)/\mathrm{E}+\mathrm{Var}\left(\mathrm{E}\right)/\mathrm{E}\mathrm{xR}\right) $$

Pearson’s correlations among all pairs of environments of GY was calculated based on genotype performance of each experimental genotype for each environment using R software package “corrplot”. The GGE Biplot which describes the relationship between different environments was performed using GEA-R (Genotype x Environment Analysis with R for Windows) Version 4. 1 [47]. The population structure (Q matrix) for the F_3:6_ Nebraska winter wheat was performed using the criteria described in [24]. The analysis was done by STRUCTURE 3.4.0 [49] and the kinship matrix (K) was estimated using TASSEL v5.2.40 [12].

### Climate data analysis

The monthly average temperature, average rainfall and average snowfall were collected from (https://www.usclimatedata.com/climate/united-states/us). Principal component analysis was done for all climate factors using ClustVis online tool. This web server is freely available at http://biit.cs.ut.ee/clustvis/ [43]. The scatter plot was visualized using excel 2016.

### Genome-wide association studies

The GWAS analysis was conducted separately for GY at each environment using 11,991 SNPs markers after filtration to remove SNPs with minor allele frequencies (MAF < 0.05) and exclude all the heterozygous SNPs which were calculated as missing values. The GY phenotypic values, Kinship matrix, Q matrix and SNPs were subjected to association analysis using a – mixed linear model (MLM) in TASSEL v5.2.40 software.

The –log^10^ *P-*values of the MLM were later adjusted by calculating the corresponding Bonferroni correction (BC) at a significance level of 5%. Phenotypic effects at the marker loci were calculated as differences between the means of the marker classes. The phenotypic variance explained (R^2^) by significant makers was determined using TASSEL v5.2.40.

Manhattan plots for grain yield trait were visualized using the Shiny AIM application [31]. Linkage disequilibrium (*r*^*2*^) was estimated using TASSEL 5.0 between each pair of SNPs located on the same chromosome. The LD heatmap was visualized using ‘LDheatmap’ R package [57].

### Candidate genes linked with grain yield

The physical position of high LD genomic regions that include the significant SNPs were used to identify the high-confidence (HC) putative candidate gene models using annotations version provided by the IWGSC. We used the recently published wheat genome sequence. WheatMine web-based platform, was used to identify the gene annotations and gene ontologies for the potential candidate genes based on IWGSC v1.0 and v1.1 (https://urgi.versailles.inra.fr/WheatMine/begin.do).

## Results and discussion

### Effects of environment (E), genotype (G), and G × E interaction (GEI)

The ANOVA for grain yield (Table 1) identified highly significant differences among genotypes at *P* < 0.0001. That high genetic variation existed among genotypes is very useful for wheat breeders to efficiently select the highest yielding genotypes, in each location or across locations, to be used in breeding programs. Genotype (G) × environment (E)interactions (GEI)were significant at *P* < 0.0001for grain yield. Significant GEI indicated that the genotypes performed differently in different environments and that genotypes should be selected for adaptation to specific environments [3, 4, 65]. Hence, the GEI confirmed that genotypes responded differently to the variation in environmental conditions at locations, which indicated the need to test wheat cultivars at multiple locations.

**Table 1:** Variance component and associated standard error estimated using a general linear mixed model by residual maximum likelihood (REML) for grain yield measured across eight locations in Nebraska and one location in Kansas in 2017

	Variance Estimate	Standard Error	Significance
**Environment (E)**	**249.19**	**124.76**	***P*** **< 0.0001**
**Iblock (Environment)**	**8.68**	**1.71**	***P*** **< 0.0001**
**Genotype (G)**	**12.80**	**1.77**	***P*** **< 0.0001**
**Genotype × Environment (GEI)**	**27.25**	**3.74**	***P*** **< 0.0001**
**Error**	**38.75**	**3.34**	***P*** **< 0.0001**
**Heritability In broad sense (H**^**2**^**)**	**0.64**		

Significance testing performed using likelihood ratio test

**Fig. 1:**
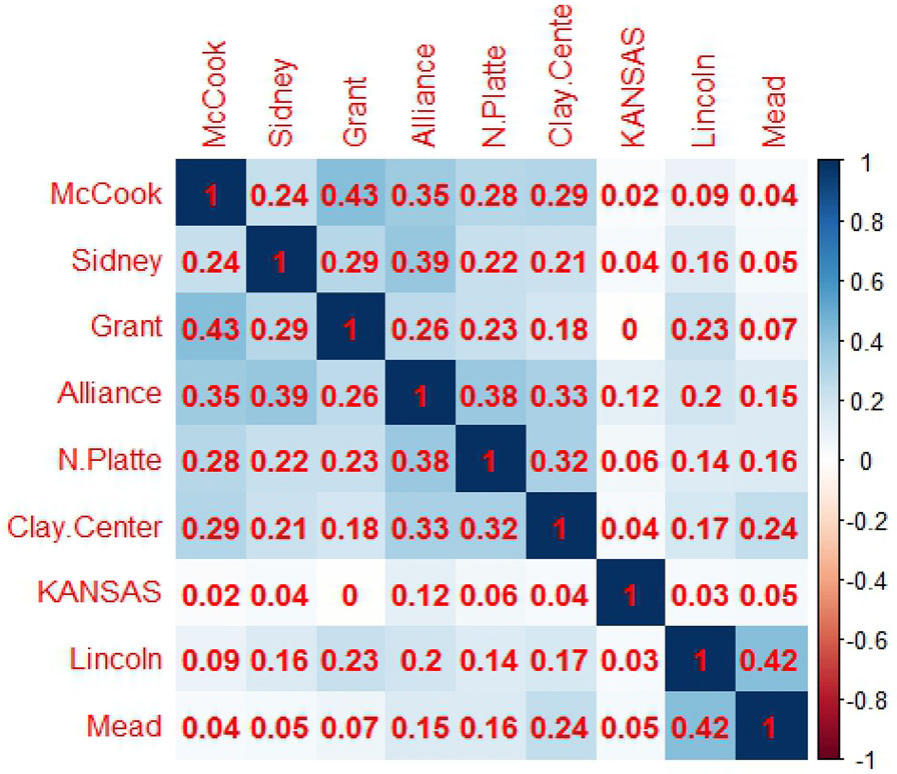
Correlation coefficient matrix of Grain yield in all nine environments

The phenotypic correlation for grain yield among the nine environments is presented in (Fig. 1). No significant or very low significant correlations at *P* < 0.05 were observed among the cultivar yield values in all environments. A moderately positive significant correlation between Lincoln and Mead (*r* = 0.42*) and between Grant and McCook (*r* = 0.43*) was expected as these pairs of locations are in similar ecogeographic zones. The results of weak or low correlations further support the diversity of the testing environments and the significant effect of GEI on the genotypes’ performances.

### The performance of the genotypes in different environments

It is common for MEYTs data to represent a combination of crossover and non-crossover types of GEI. The minimum, maximum, mean of grain yield in each environment is presented in Table 2. The maximum grain yield ranged from 3503.53 (Kansas) to 8287.50 Kg/Ha (McCook). The lowest and highest average of grain yield were also accounted to the same two environments. This huge difference in grain yield for the same set of genotypes was due to the strong effect of environment and GEI.

**Table 2:** The Maximum, minimum and mean of grain yield trait (Kg/Ha) measured across eight locations in Nebraska and one location in Kansas in 2017

Environments	Max	Min	Mean
Alliance	4737.57	2683.52	3688.10
Clay Center	5472.09	3169.01	4377.17
Grant-D	3796.24	2509.37	3213.25
Kansas	3503.53	1663.30	2543.39
Lincoln	5676.86	2710.02	4289.65
McCook	8287.50	4952.45	6044.39
Mead	4135.17	1628.13	3046.68
North Platte	3915.26	2644.65	3281.68
Sidney	3850.33	3342.43	3608.08

The highest yielding genotypes differed by location; NE17660 (Alliance), NE17626 (Clay Center), NE17528 (Grant), NE17588 (Kansas), NE17609 (Lincoln), NE17441 (McCook), NE17662 (Mead), NE17463 (North Platte), and NHH17447 (Sidney) (Supplementary Table S3). GEI can be caused by crossover interactions or by non-crossover interactions (e.g. changes in the magnitude of the differences among lines). As there was no common genotype that ranked as the highest yielding genotype in more than one environment, we chose the 50 highest yielding genotypes (~ 18.5% of the experimental genotypes) in each location to represent the high yielding genotypes at that environment. Then, a genotype was selected if it was among the 50 high yielding genotypes in at least two environments. As a result, 13 genotypes were marked and selected (Table 3). The same procedure was applied in selecting the high drought tolerant wheat genotypes, [52]. Those genotypes which were in the high yield group in multiple locations were considered as having the non-crossover GEI (Table 3). The genotype NE17625 was found among the highest 50 yielding genotypes in all the environments except Kansas. The genotype NE17626 was found among the highest 50 yielding genotypes at all the environments except Mead. Moreover, the genotype NE17443 was found to be among the highest 50 yielding in seven environments. Two genotypes NE17629 and NE17549 were found among highest 50 yielding in six environments. Remarkably the selected genotypes are heterogeneous in terms of their pedigree. For example, some of the selected genotypes shared the same parent such as NE17625, NE17626, NE17629 and NE17549 which were all reselections from NW03666 (Supplementary Table S1). Both NE17479 and NE17435 were half-sibs and shared the same parents (NE06545/NW07534). The other seven selected genotypes had different pedigrees. Pedigree information provides useful information for plant breeders to maintain diversity while making the next set of crosses using the selected genotypes.

**Table 3:** The best high yielding genotypes across all environments

Genotypes/Env.	Alliance	Clay Center	Grant	Kansas	Lincoln	McCook	Mead	North Platte	Sidney	Total
NE17625	*	*	*		*	*	*	*	*	8
NE17626	*	*	*	*	*	*		*	*	8
NE17443	*	*	*	*	*			*	*	7
NE17629		*	*	*		*	*	*		6
NE17549	*	*	*			*	*	*		6
NE17435	*	*			*	*	*			5
NE17479	*		*	*	*			*		5
NE17524		*	*		*	*	*			5
NE17533	*			*		*	*	*		5
NE17545	*		*	*		*			*	5
NE17661	*		*			*		*	*	5
NE17550	*	*	*		*				*	5
NE17624	*		*		*	*	*			5
Total	11	8	11	6	8	10	7	8	6	

As mentioned previously, the significant GEI often is interpreted as a specific breeding program for improving grain yield may be required optimal improvement for each environment [44, 52]. However, crossing these selected genotypes may be useful for a breeding program, with a full consideration to the pedigree information, that extends across more than one environment, especially when the environment at a location will change from year to year (e.g. may not be predictable).

Alliance and Grant had the highest number of selected top 50 genotypes in common with 11 in each. Kansas and Sidney, on the other hand, had the fewest common selected genotypes (six genotypes). This result may be due to the Kansas trial was considerably further south and in a different state while the other eight environments are in Nebraska and where the breeding program targets its new cultivars.

Analytical approaches to GEI analysis are important for enhancing the value of MEYTs and gaining an understanding of causes of GE interactions [61, 65, 68]. The methods used to understand GEI include the characterization of trial sites according to environmental factors, using either direct measurements, calculated indices, or variables derived from crop growth models [18]. The highly significant GEI were explained by the differences in the precipitation, snow cover, and temperature from one location to another location during the growing session (Supplementary Table S4). Although eight environments are geographically within Nebraska, the climate data differed by the environment.

### GGE bi-plot analysis

**Fig. 2:**
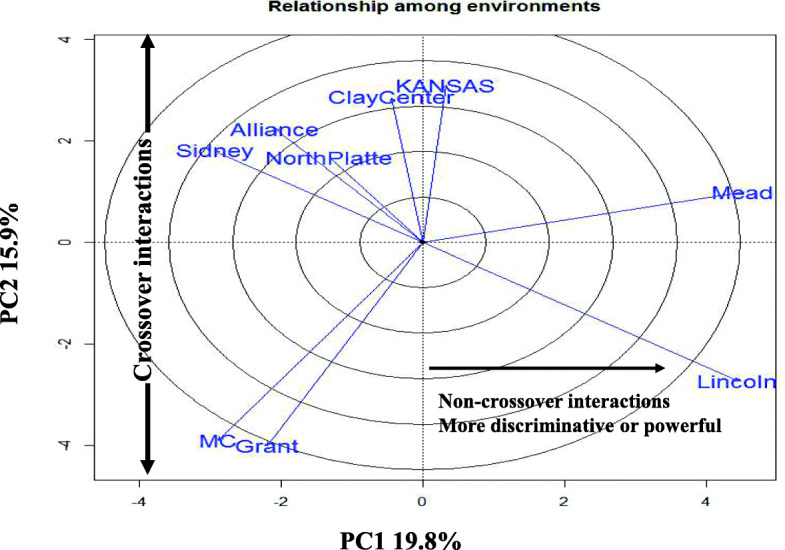
GGE-biplot based on environment-focused scaling for environments. PC and E stand for principal component and environments, respectively. Details of environments are (Supplementary Table S4). The environments are represented in this figure as Alliance (AL), Clay Center (CC), Grant (G), Kansas (KAN), Lincoln (LN), McCook (MC), Mead (ME), North Platte (NP), Sidney (SD)

The GGE-biplot approach, which was based on environment focused scaling, was used to estimate the relationships between the environments (Fig. 2). The lines that join the biplot origin and the markers of the environments are called environment vectors. The angle between the vectors of 2 environments is related to the correlation coefficient between them. The angles among most of our environments were only a little smaller than 90°; therefore, the correlation between them should be close to 0 (See Fig. 1). This GGE biplot approach (Fig. 2) suggested that Alliance and North Platte were the most closely correlated environments with Grant and McCook closely behind. However, the largest correlation coefficients were between McCook and Grant and between Lincoln and Mead (Fig. 1). Some contradictions between the figures and actual correlations were predictable because the biplot did not estimate 100% of the GGE variation [33, 67].

Most of our environments in this study were considered as PC2 environments except Kansas, Lincoln and Mead were included in PC1, which had positive and negative scores. PC1 represents proportional genotype yield differences across environments, which leads to a non-crossover GEI. Genotypes with superior PC1 scores can be easily identified in environments with larger PC1 scores. In contrast to environmental PC1, PC2 had both positive and negative scores (Fig. 2). Positive and negative scores are due to crossover GEI, leading to inconsistent genotype yield differences across environments [66]. A genotype may have large positive interactions with some environments; but have large negative interactions with other environments.

**Fig. 3:**
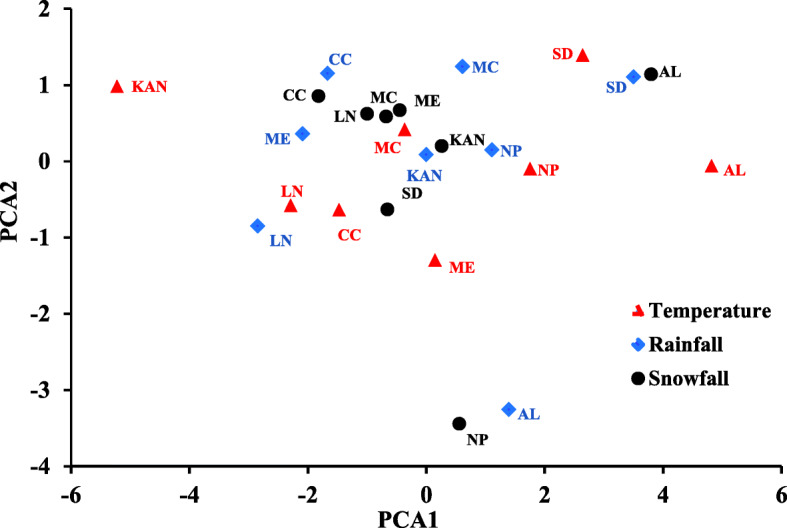
Principal component analysis for temperature, rainfall, and snowfall in the nine environments. The environments are represented in this figure as Alliance (AL), Clay Center (CC), Kansas (KAN), Lincoln (LN), McCook (MC), Mead (ME), North Platte (NP), Sidney (SD). Weather data in Grant location is not availbe

In order to create a detailed climate factor (Supplementary Table S4 and Fig. 3), PCA evaluated the standardized values of the growing season mean temperature, average rainfall and average snowfall. Looking at (Fig. 3) we find that all the three climatic factors (average temperature, average rainfall, and average snowfall) were widely distributed throughout the PCA1 and PCA2. But there were several closely observed snowfall points among Lincoln, Mead, McCook and Kansas. The average temperature of Alliance, Sidney and Kansas were widely distributed across the PCA. All these informative data indicated that different climate factors caused strong GE interactions.

### Genome-wide association study for grain yield

**Fig. 4:**
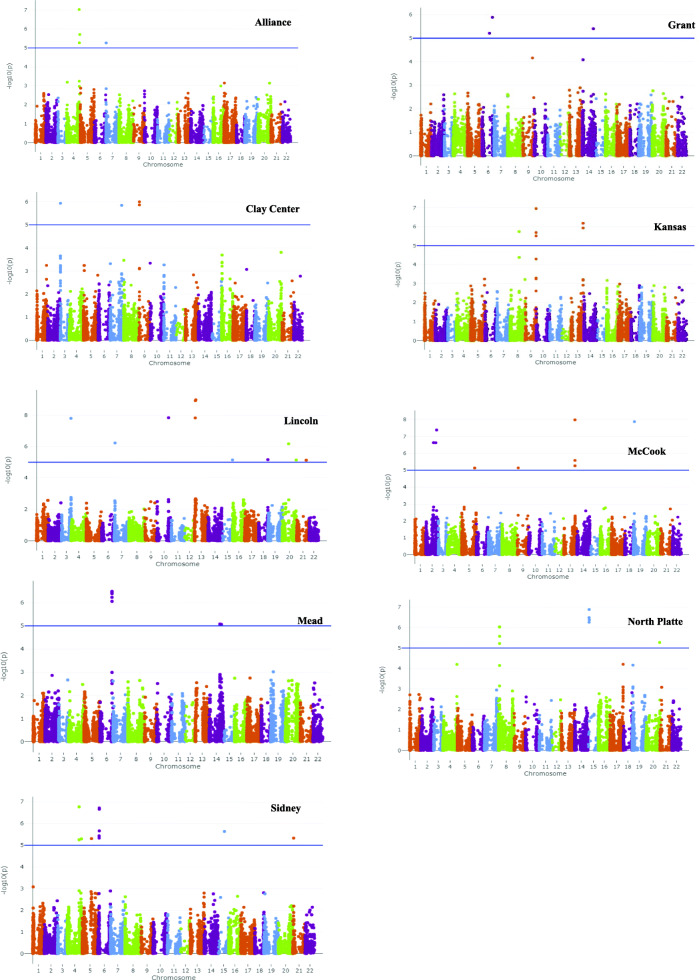
Manhattan plot displaying SNP markers-trait association identified for GY traits in GWAS using F_3:6_ Nebraska winter wheat. The blue line is significant threshold of 5% bonferroni correction (BC 5%)

**Fig. 5:**
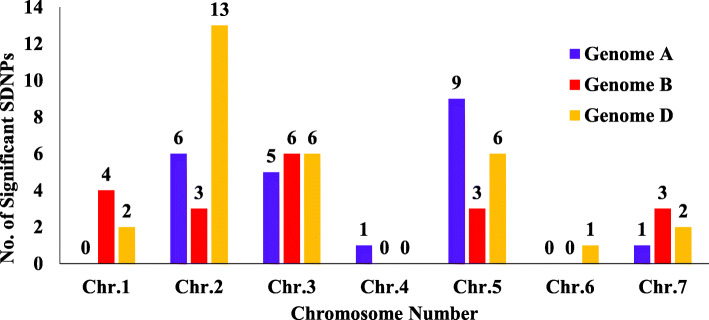
The distribution of significant associated SNPs with GY across all environments

The GWAS analysis was performed using MLM model which takes population structure into consideration [6, 69]. Due to the highly significant interaction among the genotypes and environments, the GWAS was performed for each environment, separately. The GWAS found a total of 70 MTAs associated with GY in the nine environments (Table 4 and Figure 4; Supplementary Table S5). The lowest number of significant SNPs (three SNPs) for grain yield were detected in the Grant environment, while the highest number of significant SNPs (11) was observed in three environments: Lincoln, McCook, and Sidney. The phenotypic variation (R^2^) ranged from 7.36% to 12.91%. All QTLs detected using GWAS can be considered as having minor effects on increasing grain yield. Grain yield is a complex trait controlled by many genes and affected by environment, and thus the identification of large number of associations is expected.At the genomic level, the highest number of significantly associated SNPs was observed in the D genome (30 SNPs) followed by A genome (21 SNPs) then B genome (19 SNPs) (Fig. 5). At the chromosomal level, the 71 significant SNPs associated with increased GY were distributed on all wheat chromosomes except 1A, 4B, 4D, 6A and 6B. The highest number of significant SNPs were located on the same chromosome (2D) and associated with high grain yield across environments (13 SNPs). The 13 SNPs were found in 3 environments (2 SNPs in Grant, 6 SNPs in Mead and 5 SNPs in Sidney). These valuable results reflected the importance of D genome in the GY traits. A broad comparison of marker-trait association results from the current study with two previous studies were made using a chromosome basis because of differences in marker type and marker positions on different genetic maps. Edae [21] detected a stable QTL for grain yield on chromosome 2DS both under irrigated and rain-fed conditions using DArT markers. Also, the DArT marker wpt6531 on the short arm of chromosome 2DS, which was associated with yield is about 8 cM away from the wpt4144 marker, which was associated with grain yield in a previous study by Burguen et al. [14]. Previous studies have emphasized the importance of the D genome for grain yield using different types of markers [14, 20, 21, 23, 34].

**Table 4:** Summary of GWAS analysis for grain yield including number of SNPs, range of *P* value, range of R^2^, and range of allele effects in the nine environments

Environments	Number of SNPs	Log ^10^ P- Value		Range of R^2^		Range of Allele effect	
		Min	Max	Min R^2^	Max R^2^	Min allele effect	Max allele effect
Alliance	5	9.43E-08	5.44E-06	11.50	12.74	1.65 (A and C)	4.29 (T)
Clay Center	5	9.93E-07	1.43E-06	10.6	11.68	2.60 (G and T)	4.48 (G)
Grant	3	1.31E-06	6.19E-06	8.66	9.93	1.87 (C)	2.26 (C)
Kansas	7	1.11E-07	3.07E-06	10.55	12.91	1.65 (A)	2.53(G)
Lincoln	11	1.03E-09	7.38E-06	7.36	10.44	3.04 (A)	10.58 (A)
McCook	11	1.03E-08	7.39E-06	8.72	11.58	2.36 (C)	5.19(A and C)
Mead	8	3.23E-07	8.60E-06	9.53	12.28	3.24 (T)	4,25(A and C)
North Platte	9	1.32E-07	6.05E-06	9.06	11.83	2.06 (A)	3.59 (A)
Sidney	11	1.69E-07	5.60E-06	8.89	12.73	0.35 (T)	1.00 (T)

No common markers were found among environments due to the lack of or very low significant correlations among environments for grain yield. Marker-assisted selection (MAS) can be useful for specific environments. The MTAs found in this study should be validated in additional environments and germplasm before using them in MAS. Previous studies identified SNP markers associated with GY on various chromosomes (1D, 1B, 2A, 3B, 4A, 5A, 5B, 5D, 7A, and 7B) [2, 10, 21, 32, 38, 63]. Chromosomes 3B, 5A, 5B and 7A were identified as having important yield QTL using 567 loci including RFLP, SSR, and AFLP markers [58]. El-basyoni [22] identified QTLs associated with GY in different environments on chromosomes 1B, 2A, 3A, 4A, 5A, 5B, 6A, 6D and 7B using DArT markers with a previous duplicate nursery lines of Nebraska winter wheat. Moreover, significant markers associated with GY were found on chromosomes 1B, 2A, 3A, 4A, 5A, 5B, 6A, 6D, and 7B in European winter wheat [11, 17, 19, 26, 54, 55, 58, 73]. Neumann [46], detected significant markers for GY in winter wheat on the chromosomes, 3A, 3B, 7A, 5B and 7B. Markers responsible for GY were identified on chromosomes, 4B and 7D which were reported in bi-parental QTL analyses [2, 11, 17]. Significant markers associated with GY on chromosome 2A were reported in previous studies [1, 27–29, 38, 60, 62]. A new publication of Kan et al. [32] who revealed a major quantitative trait locus (QTL) QYld.osu-1BS for grain yield in 260 **F**_**2:4**_ winter wheat population of doubled haploid (DH) lines derived from the cross of Duster and Billings and they validated the QTLs using kompetitive allele specific PCR (KASP) markers for the unique sequences for QYld.osu-1BS allele.

### Linkage disequilibrium (LD) and gene annotation

The significance of linkage equilibrium (r^2^) was estimated between each pair of SNPs located on the same chromosome in each environment (Supplementary Table 5). All SNP pairs that had a high LD were considered a genomic region (GR). As a result, a set of 16 genomic regions associated with increased grain yield were identified across the nine environments. All SNPs located on the same chromosome had a high significant LD in Clay Center (two GRs), Kansas (three GRs), and North Platte (two GRs). In Alliance, there was no significant LD among SNPs located on 2A chr., while the SNPs located on 3A chr. were in significant LD. The five SNPs that are located on 2D chromosome in Mead were in a high significant LD. Among the SNPs located on the same chromosome, there were some cases which some SNP pairs had a significant LD, while the other did not have. For example, in McCook, there was a non-significant LD among SNPs on 1B chr., however, only S1B_427530781 and S1B_427530781 were in a complete LD. Likewise, in Sidney, the five SNPs located on 2D chromosomes had two groups in which SNPs were in high significant LD. The first group consisted of two SNPs (S2D_67556531 and S2D_69503850), while the second one consisted of three SNPs (S2D_76749441, S2D_77122291, and S2D_77122292). There was no significant LD between the two groups. Such information is very important to know which SNPs, located on the same chromosome, could be inherited together or individually. The significant SNPs located on the same chromosome, if the LD value among a group of target SNPs is high, then these SNPs could represent the same QTL and inherited together. If the LD is low, on the other hand, then the two significant SNPs represent two different QTLs [6, 51]. The gene annotation was identified for all genomic regions with significant LD. The candidate genes within the 16 GRs are listed in (Supplementary Table S6).

**Fig. 6:**
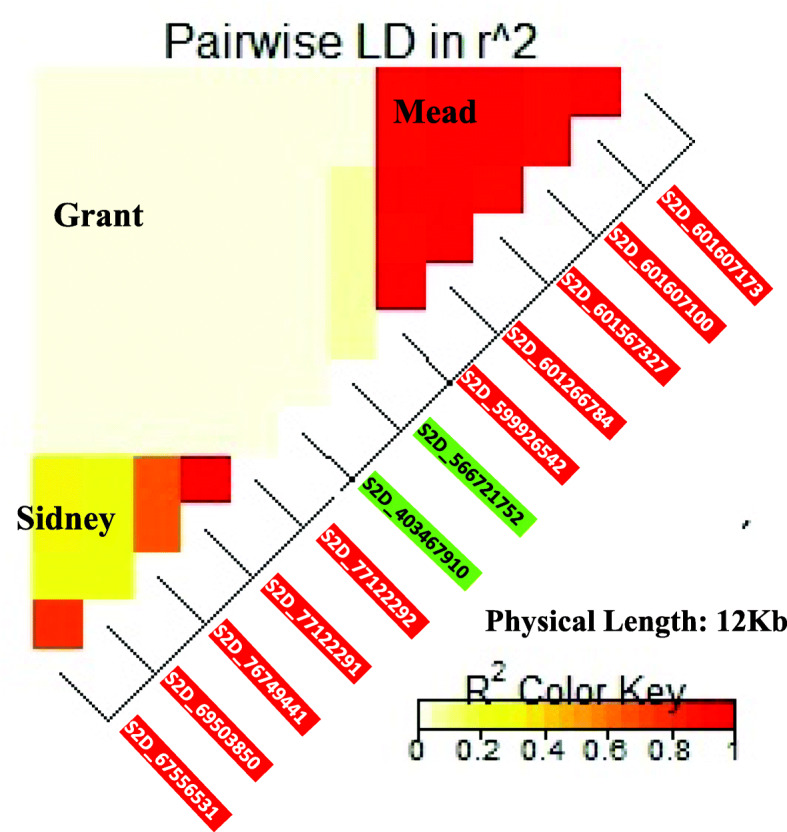
Heatmap of LD among SNPs located on 2D chr. Across the three environments. The red line indicates that high LD among all SNPs in Mead

As aforementioned, 2D chromosome had the highest number of SNPs associated with high grain yield across environment. Therefore, gene annotation was described in detail on this chromosome. By studying LD pattern among these SNPs, we can identify how many genes within the physical position of these SNPs could represent. In Mead, the five SNPs represent one QTL, while, in Sidney, S2D_67556531 and S2D_69503850 coinherited tougher and the other three SNPs on the same chromosome co-inherited together. When estimating the LD between each two pairs of the 13 SNPs, the results indicated no significant LD between any pair of SNPs from different environments (Fig. 6). This is also a further indication of the strong G × E interaction which affects the expression of genes in response to the environment.

By looking to the gene annotation in the LD genomic regions on 2D chromosome (Supplementary Table S6). We found that *TraesCS2D01G506200* gene (genome region 8) is annotated as Zinc transporter and metal ion transport was associated with grain yield in Mead environment. There was many reports demonstrated the crucial role of Zn in improving grain yield in wheat [35, 40]. Recently, Alqudah et al. [5] found that deficiency of metal ion transport specially Cu and Zn may result in reducing grain yield through increasing spikelet and floret sterility/abortion. Interestingly, in Sidney environment, *TraesCS2D01G118400* and *TraesCS2D01G119200* (genomic region 15) genes are also annotated as potassium transporter and calcium exchanger respectively, demonstrating the role of the metals in grain yield. Potassium and calcium deficiency can significantly reduce crop yield but due to the complex relationship in absorption or transport among them, their mechanisms in grain improvement is still not well-understood [56]. Other interesting candidate genes in Mead environment are involved in phytohormone e.g. auxin (*TraesCS2D01G506900*) and Jasmonate (*TraesCS2D01G507200*). In wheat, phytohormones conttrol the spikelet’s and grain development and grain filling. Slow grain development and filling rates are highly linked with low contents of the cytokinin and auxin [64] that in turn reduce grain yield. Understanding the mechanisms of metal transport and phytohormones which are found to be highly associated with grain yield in the current study are needed to improving wheat grain yield.

### The promising high yielding genotypes for future breeding program

For the 13 selected genotypes (Table 3), three criteria may be considered to determine the genotypes as candidate parents for a future cross to improve grain yield. These criteria are based on:

1. the presence of favorable alleles associated with increased grain yield was recorded in each genotype (Supplementary Table S7). NE17435 had the highest number of favorable alleles associated with increased grain yield at 46 sites, while, NE17550 had 39 favorable alleles. Four genotypes; NE17545, NE17626, NE17524, and NE17629 had the same number of 43 alleles associated with increased grain yield. Although NE17435 had the highest number of target alleles for grain yield, but it was among the 50 highest yielding genotypes in only six environments. NE17625 and NE17626 which were among the 50 highest in eight environments had 40 and 43 target alleles for grain yield, respectively. It was useful to count the number of favorable alleles that each selected genotype carries as it helps in determining the target genotypes as parents for future crosses in the breeding program to improve grain yield in single or specific environments.
2. the genetic diversity among the 13 genotypes. The GD among all genotypes in this study was extensively described in [24]. This population was divided into three possible subpopulations [24]. The genetic distance among the 13 selected genotypes is presented in (Supplementary Table S8). Five genotypes were found to be assigned to subpopulation 1 (G1), five were assigned to subpopulation 3 (G3), and the remaining three genotypes were assigned to subpopulation (G2). The highest genetic distance was found between NE17624 and NE17661 (GD = 0.333), while the lowest GD was between NE17549 and NE17625 (GD = 0.007). Generally, all selected genotypes had a low level of genetic distance and they tend to be genetically similar. This is due to the fact that all genotypes represent Nebraska winter wheat breeding program [24] Moreover, many accessions were reselections from the same line (Supplementary Table S1).
3. the number of unique alleles associated with increased grain yield between each pair of the 13 selected genotypes (Supplementary Table S9). The highest number of different alleles (33) was found between NE17549 and both NE17479 and NE17435. Only three different alleles, on the other hand, were found between NE17549 and NE17625. The different alleles between each two genotypes depends on the genetic distance among them. There was a positive significant correlation between different alleles and genetic distance (*r* = 0.65**).

Each criterion identified different candidate parents. By considering all criteria together with the priority for the number of different alleles and genetic distance, a cross between NE17625 and NE17479 may be useful for developing a cultivar that may be high yielding in five environments (Alliance, Grant, Kansas, Lincoln, and North Platte). Both parents had 40 alleles associated with increased grain yield, 32 different alleles associated with increased grain yield, and genetic distance of 0.292. Moreover, NE17625 and NE17479 were among the highest 50 genotypes in eight and five environments, respectively. Although NE17625 and NE17626 were among the highest 50 yielding genotypes in eight environments, crossing between them is not useful because it will lead to very low genetic variation in grain yield as both genotypes tend to be genetically similar with only GD of 0.007 and nine different alleles. Therefore, using genomic tools (e.g. identifying number of target alleles, number of different alleles, genetic diversity analyses, etc.) in parallel with phenotypic selection is very fruitful to improve target traits. Moving forward such information on important MTAs can be considered along with genomic selection which is now routinely performed in the breeding program for shortlisting promising parental lines for new sets of crosses for improving grain yield [9].

### Putative SNP markers for validation and future use in MAS

For the candidate SNPs detected by GWAS, a set of 10 SNP markers were found to be present in the 13 selected genotypes, while three SNPs were not shown in any of the selected genotypes. As it is recommended in each environment to validate the SNPs that were found in the that environment, it may be useful to convert these 13 SNP to kompetitive allele specific PCR (KASP) markers and validate them for high grain yield in a different genetic background. For example, S2A_718916923 marker was found in all selected genotypes and should be validated. Although the marker allele C was found to be significantly associated with increased grain yield in Alliance, this allele was also present in all selected genotypes in other environments. This marker allele has probably minor effects in increasing grain yield in the other environments but the GEI hinders this marker in the other environments to be significantly associated with grain yield.

Interestingly, two SNPs associated with GY in this study were also found to be associated with grain yield in a previous study [10]. This earlier study evaluated grain yield for synthetic winter wheat genotypes in Turkey for two seasons. The two SNPs (S3A_24993796 and S3A_24993797) are associated with GY in Alliance environment (Supplementary Table S5). Moreover, in their study, the allele A for S3A_24993796 and C for S3A_24993797 were associated with increased grain yield. The same two alleles were also associated with increased grain yield of Nebraska winter wheat. The amount of phenotypic variation (R^2^) explained by these markers is similar (~ 11.5%) in both studies. High LD between the two SNPs has also been highlighted in both of our studies. Overall, the two SNPs are associated with grain yield in two independent genetic backgrounds (Nebraska wheat and synthetic winter wheat from Turkey) and two testing environments (Nebraska and Turkey) further validating the associations identified in this study. It is known that states in the Great Plain and Turkey share some of the same climatic features [7]. Hence, introgression of favorable alleles across germplasm which have not have been previously incorporated into their germplasm may be a possibility [7].

## Conclusion

G × E interaction needs to be investigated for identifying genotypes that can be used for improving grain yield in all tested environments. However, utilizing the advances in DNA sequencing and genetic analysis by GWAS has helped in identifying possible candidate genotypes to be used in improving grain yield in a group of environments. Three criteria were suggested to identify candidate genotypes to be used as parents for crossing in a group of tested environments. These three criteria depend on combining the information at phenotypic and genomic levels. This research provided a framework for considering how to select parents for future crosses.

## Supplementary Information

**Additional file 1: Table S1.** Pedigree information for F _3:6_ Nebraska Duplicate Nursery winter wheat. **Table S2.** List of SNPs generated from GBS data. **Table S3.** The genotypes performance for GY under nine environments. **Table S4.** Average high and low monthly temperatures, total monthly precipitation, and total snowfall in all environments except Grant and Kansas for growing season (2016 /2017). **Table S5.** Details of GWAS analysis for grain yield using mixed linear model (MLM) at the significance level of 5% bonferroni correction using 11,991 SNPs. **Table S6.** gene annotation and candidate genes for the high LD genomic regions.** Table S7.** The presence (1) and absence (0) of all alleles associated with high yielding detected by GWAS in 13 selected genotypes in all environments. **Table S8.** The distance matrix based on genotypic data between the best high yielding genotypes. **Table S9.** The different allele effects matrix among the 13 selected genotypes.

## Data Availability

The datasets generated and/or analyzed during the current study are available in the NBCI repository, http://www.ncbi.nlm.nih.gov/bioproject/680548

## Abbreviations

AFLPs: Amplified fragment length polymorphisms
ANOVA: Analysis of Variance
CIMMYT: The international Maize and Wheat Improvement Center
DUP17: Nebraska Duplicate Nursery 2017
GBS: The genotyping-by-sequencing
GEI: Genotype × environment interaction
GRs: Genomic regions
GY: Grain yield
GWAS: Genome-wide association study
KASP: Kompetitive allele specific PCR
LD: Linkage disequilibrium
MEYTs: Multi-environment yield trials
MTAs: Marker Trait Association
PCA: Principal component analysis
QTls: Quantitative trait loci
RFLPs: Restriction fragment length polymorphisms
RMEL: The residual maximum likelihood
SNPs: Single nucleotide polymorphisms
SSRs: Simple sequence repeats
